# Pharmacological Validation for the Folklore Use of *Ipomoea nil* against Asthma: In Vivo and In Vitro Evaluation

**DOI:** 10.3390/molecules27144653

**Published:** 2022-07-21

**Authors:** Taha Alqahtani, Sajida Parveen, Yahia Alghazwani, Hanan M. Alharbi, Reem M. Gahtani, Nadia Hussain, Kashif ur Rehman, Musaddique Hussain

**Affiliations:** 1 Department of Pharmacology, College of Pharmacy, King Khalid University, Abha 62529, Saudi Arabia; ttaha@kku.edu.sa (T.A.); ysghazwani@kku.edu.sa (Y.A.); 2 Faculty of Pharmacy, TheIslamia University of Bahawalpur, Bahawalpur 63100, Pakistan; zsajida97@gmail.com (S.P.); kashifur.rahman@iub.edu.pk (K.u.R.); 3 Department of Pharmaceutics, College of Pharmacy, Umm A-Qura University, Makkah 21955, Saudi Arabia; hmsharbi@uqu.edu.sa; 4 Department of Clinical Laboratory Sciences, College of Applied Medical Sciences, King Khalid University, Abha 61421, Saudi Arabia; rmalqahtani@kku.edu.sa; 5 Department of Pharmaceutical Sciences, College of Pharmacy, Al Ain University, Al Ain 64141, United Arab Emirates; nadia.hussain@aau.ac.ae

**Keywords:** *Ipomoeanil* seeds, bronchodilator, anti-asthmatic, IgE antibody, inflammatory cells, hyper-responsive

## Abstract

Oxidative stress is the key factor that strengthens free radical generation which stimulates lung inflammation. The aim was to explore antioxidant, bronchodilatory along with anti-asthmatic potential of folkloric plants and the aqueous methanolic crude extract of *Ipomoea nil* (*In.Cr*) seeds which may demonstrate as more potent, economically affordable, having an improved antioxidant profile and providing evidence as exclusive therapeutic agents in respiratory pharmacology. In vitro antioxidant temperament was executed by DPPH, TFC, TPC and HPLC in addition to enzyme inhibition (cholinesterase) analysis; a bronchodilator assay on rabbit’s trachea as well as in vivo OVA-induced allergic asthmatic activity was performed on mice. In vitro analysis of 1,1-Diphenyl-2-picrylhydrazyl radical (DPPH) expressed as % inhibition 86.28 ± 0.25 with IC_50_ 17.22 ± 0.56 mol/L, TPC 115.5 ± 1.02 mg GAE/g of dry sample, TFC 50.44 ± 1.06 mg QE/g dry weight of sample, inhibition in cholinesterase levels for acetyl and butyryl with IC_50_ (0.60 ± 0.67 and 1.5 ± 0.04 mol/L) in comparison with standard 0.06 ± 0.002 and 0.30 ± 0.003, respectively, while HPLC characterization of *In.Cr* confirmed the existence with identification as well as quantification of various polyphenolics and flavonoids i.e., gallic acid, vanillic acid, chlorogenic acid, quercetin, kaempferol and others. However, oral gavage of *In.Cr* at different doses in rabbits showed a better brochodilation profile as compared to carbachol and K^+^-induced bronchospasm. More significant (*p* < 0.01) reduction in OVA-induced allergic hyper-responses i.e., inflammatory cells grade, antibody IgE as well as altered IFN-α in airways were observed at three different doses of *In.Cr*. It can be concluded that sound mechanistic basis i.e., the existence of antioxidants: various phenolic and flavonoids, calcium antagonist(s) as well as enzymes’ inhibition profile, validates folkloric consumptions of this traditionally used plant to treat ailments of respiration.

## 1. Introduction

Respiratory structure is emphasized as the foremost operating network in active bodies. Instabilities in its histo-physiological features and other characteristic alteresphysio-anatomical forms and a pathological state are settled i.e., inflammational airways, presenting allergic asthma, acute lung injury (ALI), broncho-alveolar hyper exudations emerging COPD [1,2,3] or former prolonged respiratory ailments, lethal to an entity. Airway inflammation can be produced due to pollen, filth, allergens [4], xenobiotics or any pathogen as a result of oxidative stress by reactive oxygen species production. During this process, pro-inflammatory as well as inflammatory cells generate and cause allergic acute and chronic inflammation of lungs, kidney, heart and neurodegenrative problems by altering the balance between the oxidative process and antioxidant level in the body [5,6]. Allergic asthmatic state is airway over activity and intensification in phlegm secretion. T-Helper cells, i.e., type 1 and type 2, interleukins (IL-4, IL-5, IL-13) [7], NF-κB [8], chemokine, less interferons, immunoglobins, play a vigorous role in the expansion of acute and/ chronic asthmatic episodes [9]. The top role of these mediators is central in asthmatic problems, as labelled in miscellaneous studies beforehand [8,10]. Epithelial cells, pseudostratified cells, dendritic cells, mast cells and mucus secreting cells are present in the airway’s lumen. Vagus sub-epithelial cells, in addition to receptors, discharge acetylcholine on bronchial muscles. Parasympathetic action cause airways to narrow, so, when this is going to antagonize, generate dilatation of airways so anti-muscarinics are used in asthma, COPD [4], and other respiratory infections [11]. Different medicines, e.g., short and/long acting beta 2 agonists, and corticosteroids have been engaged as therapeutic agents as desirable treatment plus prevention for these sorts of disorders to treat broncho-constriction, asthma and lung damage [12]. However, tenacious treatments, besides their immediate termination outcomes as side effects, may demonstrate as being lethal for the social life cycle and express necessity to sightsee traditional medicinal flora to discover new reliable along with life-saving medicinal elements [11]. *Ipomoea nil* (Linn) Roth. (*Convolvulaceae*) is known as ivy morning glory; seed usage is common, and flowers are produced from June to October. It is indigenous to Punjab and distribution is in Pakistan in addition to India [13]. Folkloric reputation as anti-inflammatory, blood purifier, anthelmintic, astringent, laxatives, anti-emetic, carminatives and is considered useful to treat a series of ailments like abdominal diseases, asthma, hypertension, disease of liver and joints, drying the phlegm [13], erectile dysfunction and for lubrication obligations. Application of seeds paste is used for cosmetic purposes i.e., dry skin, freckles, etc. In addition, it is also hepatoprotective, anti-diabetes [14,15], anti-interleukin-8 (IL-8), and anti-inflammatory [16,17]. Phytoanalysisexposed the occurrenceof alkaloids (chanoclavine [18], penniclavine, elymoclavine), α-methyl-β-oxybutyric acid, pharbitinic acid, phytoecdysone, tiglic acid, plytosteratin, lysergol [18], ecdysteriods, hederasterones, stigmasterol 3-*O*-β-D-glucoside, 20-hydroxyecdysone, β-sitosterol-3-*O*-β-D-glucoside, ethylcaffeate, anthocyanin, i.e., cyanidin, cyanidin 3-sophoroside and β-d-glucopyranoside [19]. The utmost contemporary investigated phytochemicals are hederaceterpenol, hederaterpenoside, triterpenoid plus stigmast-5-en-3-*O*-β-D-glucopyranoside. The aim of the current research effort on the aqueous-methanolic crude extract of *Ipomoea nil* seeds was commenced to observe its antioxidant, bronchodilator, anti-asthmatic and enzyme inhibition action with mechanistic approaches.

## 2. Materials and Methods

### 2.1. Plant Extraction

Dried seeds of *Ipomoea nil* were purchased from Multan market and recognized by AltafDasti (taxonomist), BZU, Multan. Plant specimen voucher, Fl. 575–08, was retained in the extract house of the Institute. About one kilogram of powdered seeds was macerated in 70% aqueous-methanol for extraction at 24 °C [20,21] for three days three times. After maceration, filtration was completed with the help of muslin cloth and Whatman-1 filter paper and evaporation subsequently [22] with 13.5% yield. It was named *In.Cr* and put in an amber glass jar in a refrigerator.

### 2.2. Chemicals

Highest purity and research grade solvents, chemicals and drugs were used in the experiments. Following chemicals and drugs, i.e., acetylcholine chloride, carbachol (Cch), potassium chloride, dicycloamine, magnesium chloride, Dexamethasone (Dex), ethylene diamine tetra-acetic acid (EDTA), QuantikineELISA kits, gallic acid, quercetinand ovalbumin (OVA) were purchased from Sigma-Aldrich Chemicals Co., St Louis, MO, USA. Calcium chloride, glucose, magnesium sulphate, potassium dihydrogen phosphate, sodium bicarbonate, dichloromethane (DCM), sodium dihydrogen phosphate, Folin–Ciocalteu reagent and methanol were obtained from Merck, Darmstadt, Germany. Ammonium hydroxide, sodium chloride, and sodium hydroxide were purchased from BDH Laboratory supplies, Poole, the United Kingdom. An ultrasonic nebulizer (NE-U12; Omron Corp., Tokyo, Japan) was purchased from Tokyo, Japan. Diff-Quik (staining reagent IMEB Inc., San Marcos, CA, USA) was obtained from the USA. A rotary evaporator (Rotavapor, BUCHI labrotechnik AG, Model 9230, Flawil, Switzerland) was used. The vehicles used for the drug solublization do not possess any effect on tissue functioning and contractility in control experiments.

### 2.3. Animals

Animals (♂/♀) used were a locally availed strain of rabbits (1.0–2.0 kg), having an age of 5–6 months and mice (Swiss albino 30–45 g) with standard food and tap water ad libitumand housed under controlled environmental conditions (25 °C) at the animal house of Faculty of Pharmacy, BZU, Multan. Food was withdrawn at least 24 h before the commencement of experiments. The rabbits were sacrificed to remove tracheas for in vitro experiments. All the animal protocols alongwith experiments were executed [23], and permission was obtained from the Animals Ethical Agency of BZU, Multan, with reference number EC/02/2021 dated 14 January 2021.

### 2.4. Phytoanalysis

Phytochemical screening of *In.Cr* tests to identify alkaloids, coumarins, anthraquinones, saponins, tannins, flavonoids and terpenes as potential foremost elements of the plant, as by [24,25].

### 2.5. HPLC Characterization

Acid hydrolysis of *In.Cr* was carried out using the previously published method by [26] through some adjustments. In short, the solution comprising *In.Cr* (50 mg) and methanol (24 mL) was made followed by addition of purified water (16 mL). Then, the solution was processed with 6M HCl (10 mL) and incubated for 2 h at 95 °C. A 0.45 µL nylon filter (Biotech, City, Germany) was used to filter the mixture before proceeding with the HPLC to avoid impurities. HPLC (SPD-10 AV Shimadzu, Japan) was performed using 250 mm × 4.6 mm, 5 µm, shim-pack CLC-ODS (C-18) column. The gradient HPLC with mobile phase; solvent A (water: acetic acid [96:4, at 2.27 pH]), solvent B (acetonitrile [100%]) was done for phenolic compounds and quercetin. The gradient was set as 0–15 min (15%B), 15–30 min (45%B) and 35–45 min (100% B) with 1 mL/min of flow rate. To separate kaempferol, the isocratic HPLC was conducted with the mobile phase acetonitrile: dichloromethane:methanol (60:20:20). A UV/visible HPLC detector was exploited at the wavelength of 280 nm, in order to determine the eluted phenolic components. The retention time and peak areas of isolated components were then matched with the reference standards to identify these components.

### 2.6. DPPH Assay

DPPH (1,1-Diphenyl-2-picrylhydrazyl radical) assay of *In.Cr* was performed by [27]. Free radical scavenging activity was resoluted by the DPPH (1,1-Diphenyl-2-picrylhydrazyl radical) assay, which was carried out by following the previously reported method [28], by using 96-well microtitreplate at room temperature. Quercetin was used as a standard drug, while DMSO was taken as negative control, and all tests were performed in triplicate. The reduction in the absorbance was measured at 515 nm using a Synergy HT BioTek^®^ USA microplate reader (Winooski, VT, USA), and data obtained were computed on Ez-fit software (Dayton Lamina Corporation, Dayton, FL, USA). Percentage radical scavenging activity was calculated by using the following formula:

% RSA = 100 − {(OD test compound/OD control) × 100}

Wherever OD test = optical density of crude extract; and OD control = optical density of control.

### 2.7. Total Phenolic Contents Determination

The total phenolic content (TPC) of the plant extract was determined by Folin–Ciocalteau’s method performed by [28] with minor modifications. Sterilized test tubes were taken and three hundred microliters of samples (ppm) were added in it. Then, 1.5 mL of Folin–Ciocalteau reagent (10× dilution) and 1.0 mL of sodium carbonate (7.5%) were added. After proper mixing, tubes were kept in the dark for 30 min. The absorbance was checked via spectrophotometer (PRIM, France) at 765 nm. TPC was expressed as gallic acid equivalents (GAE) per 100 g of renewed material.

### 2.8. Total Flavonoids Assay

Total flavonoid content was measured by AlCl_3_ colorimetric assay, executed by [29]. Briefly, 1 mg/mL of plant extract/standard solutions of quercetin (20, 40, 60 and 100 μg/mL) was added to a volumetric flask having 4 mL of dH_2_O. In addition, 0.30 mL 5% NaNO_2_ and after five minutes 0.3 mL 10% AlCl3 were added in a flask, respectively. Then, IM NaOH (2 mL) was added, and the volume was prepared up to 10 mL with distilled water. Absorbance was taken against the blank at 510 nm. TFC was articulated as mg Quercetin Equivalents (mg QE).

### 2.9. Cholinesterase Activity

Enzyme inhibition profile of *In.Cr* was resolute by the manufacturer’s instructions as in [25]. The decrease in absorbance indicates the increase in enzyme inhibition activity, which was calculated with the following equation:

Inhibition (%) = (Abs. of control − Abs. of test soln.) × 100/Abs. of control

where

Absorbance of control = total enzyme activity without inhibitor;

Absorbance of test = Activity in the presence of test compound;

IC_50_ values were evaluated using EZ–Fit Enzyme kinetics software (Perrella Scientific Inc., Amherst, MA, USA).

### 2.10. In-Vitro Bronchodilator Activity

Bronchodilator activity was performed by the method adapted by [30]. Kreb’s solution having the following composition (mM): NaCl (118.2), CaCl_2_ (2.5), NaHCO_3_ (25.0), KCl (4.7), KH_2_PO_4_ (1.3), MgSO_4_ (1.2) and glucose (11.7) were used to place the trachea of the rabbit. The trachea was cleaned and rings of 2–3 mm girth containing 2 to 3 cartilages were prepared. Each ring was unlocked by a longitudinal incision on the ventral area opposite to the smooth muscles layer to form a strip with a smooth muscles layer in the middle as well as cartilages on both sides, forming a tracheal strip with a smooth muscle sandwiched between cartilaginous portions on the edges. These tracheal preparations were suspended in isolated tissue baths containing 10 mL of the Kreb’s solution having pH 7.4, sustained at 37 °C, aerated continuously with a mixture of 95% Oxygen and 5% Carbon dioxide (carbogen). A preload tension of 1 g was applied and sustained through the whole experiment, and tissue preparations were allowed to be equilibrated for 1 h prior to application of *In.Cr* Tissue preparations were stabilized by repeated applications of Cch (1 μM) until constant responses were recorded. The Cch (1 μM)- and K^+^(80 mM)-induced sustained contractions were subsequently used for testing of different doses of the *In.Cr* in cumulative ways [31]. The responses were recorded through a power lab data acquisition system (AD Instruments, Sydney, Australia) attached to a computer with installation with lab chart software (Version 6). The relaxant effect of the *In.Cr* was assessed on Cch (1 μM)- and K^+^(80 mM)-induced contractions in isolated rabbit tracheal preparation as cumulative addition of the test material to the isolated tissue bath may relax the isolated rabbit tracheal preparation. The standard dicyclomine with Ca^2+^ channel blocking effect was tested on K^+^(80 mM)- and Cch(1 μM)-induced spastic contractions in order to confirm the possible mechanism of action.

### 2.11. In-Vivo albumin (OVA)-Sensitized Allergic Asthmatic Assay

To perform this, Ref. [32] was followed by certain modifications. Male mice were used and acclimatized for two weeks and then grouped, each with eight animals; control (normal saline, 10 mL/kg), model group (20 μg OVA, emulsified with 2 mg aluminum hydroxide in 200 μL phosphate-buffered saline (PBS) buffer), experimental/test groups (different doses of *In.Cr*) and a reference group (dexamethasone,1 mg/kg). The animals were sensitized on the 7th, 15th, 21st and 28th days. Total duration of experimentation with animals was 28 days. In addition, 100, 200 as well as 300 mg/kg of *In.Cr* were given (oral gavage) 1 h before the OVA challenge. In addition, 24 h later, BALF was collected by intra-tracheal intubation with three times infusion of ice-cold PBS (0.5 mL) into the lung and centrifuged at 4 °C for 10 min. and a hemocytometer was used for total inflammatory cell counting within (≤5 square), through staining.

### 2.12. ELISA Assay

The level of IgE in serum and concentrations of interleukins, i.e., IL-4 and IL-13 in BALF, was evaluated by adaptation of a method developed by Wei et al. [33] according to their manufacturers’ guidelines. The concentration of OVA-specific-IgE in serum was measured using sandwich ELISAs. Antibodies were detected in the serum using isotype-specific secondary antibodies. Upto 200 μL of *o*-phenylenediaminedihydrochloride was added to each well, after washes. For 10 min, plates were incubated in the dark, and the measured absorbance was at 450 nm. OVA-specific-IgE concentration was estimated from a standard curve produced using 250 ng/mL recombinant IgE.

### 2.13. Statistical Analysis

The data were showed as mean ± SEM alongside EC_50_ values. Graph pad Prism (10) (GraphPad Software, Inc., San Diego, CA, USA) was used to find DRC, and Student’s *t*-test was employed for analysis. *p* < 0.05 was measured as statistically significant.

## 3. Results

### 3.1. Phytochemical Investigation

Plant extract (*In.Cr*) was found to contain alkaloids, saponins, anthraquinones, sterols, tannins, coumarins, flavonoids and terpenes as reported by [18].

### 3.2. HPLC-Based Characterization of In.Cr

**Figure 1:**
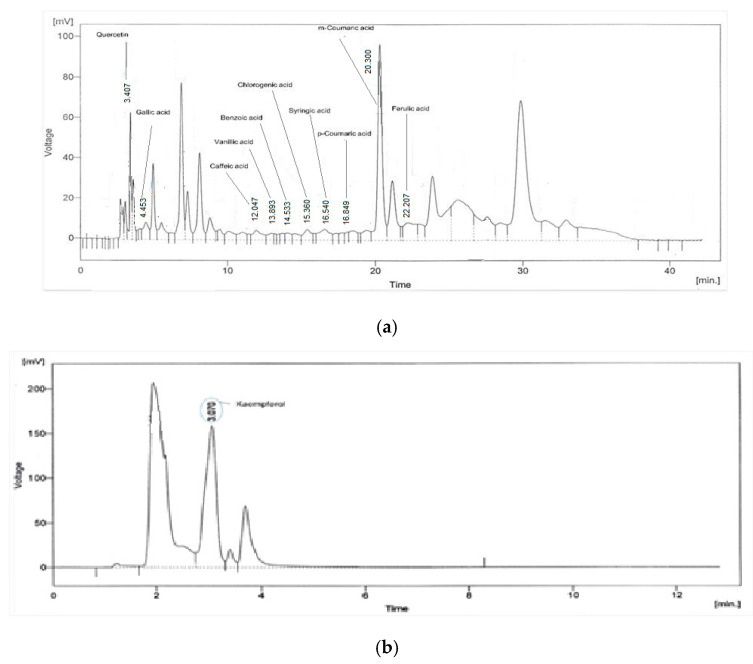
Chromatograms express (**a**) phenolic acids and (**b**) kaempferol of plant (*In.Cr*): HPLC peak areas of compounds identified, quantified and saponified by HPLC extracted from the crude extract of seeds of *Ipomoea nil*.

The HPLC is suitable for both quantitative and qualitative analysis of naturally occurring compounds. Column chromatographic characterization and quantitative modeling were performed on *In.Cr* polyphenolics and flavonoids:Kaempferol and quercetin, along with phenolic acids: synergistic acid, ferulic acid, vanillic acid, caffeic acid, coumaric acid, gallic acid and chlorogenic acid, were identified in the HPLC analysis. Figure 1a shows the chromatogham of phenolic acids and quercetin while Figure 1b shows the chromatogram of kaempferol.

### 3.3. DPPH Assay

*In.Cr* (0.5 mg/mL) exhibited scavenging activity against stable free radicals, by 1,1-Diphenyl-2-picrylhydrazyl radical (DPPH) as compared to Quercetin (0.3 mM/L) (Table 1). Quercetin was used as a standard drug while DMSO was taken as a negative control, and all tests were performed in triplicate. The reduction in the absorbance was measured at 515 nm using a Synergy HT BioTek^®^ USA microplate reader, and data obtained were computed on Ez-fit software. Percentage radical scavenging activity was calculated by using the following formula:

% RSA = 100 − {(OD test compound/OD control) × 100}

where OD test = optical density of crude extract; and OD control = optical density of control.

### 3.4. Total Phenolic Contents (TPC)

The contents of total phenols in *In.Cr* were expressed as gallic acid equivalents’dry weight of extract GAE/g. The calibration curve of gallic acid in different concentrations ranging from 10 to 50 µg/mL was used and the calculated TPC in *In.Cr* usinga standard calibration curve (Y = 0.0046 × 0.0315, R^2^ = 0.9958) found to possess 218.5 ± 2.042 as given in Table 2.

### 3.5. Total Flavonoid Contents

Total flavonoid contents were estimated by a calibration curve of standard quercetin equivalents mg QE/g of *In.Cr* (plant extract). The calibration curve of quercetin in distinct concentrations from 10 to 50 µg/mL was used and the calculated TFC in *In.Cr* using a standard calibration curve (Y = 0.0018x + 0.1063, R^2^ = 0.9726) invented to possess 139 9.47 ± 7.014 mg QE/g as given in Table 2.

### 3.6. Enzyme Reduction Profile of In.Cr

The profile of percent decrease in enzymes via *In.Cr* (0.5 mg/mL) and standard drug, and Eserine (0.25 mM/L) is described in Table 3.

### 3.7. In-Vitro Effect of In.Cr on Isolated Trachea Preparations

**Figure 2:**
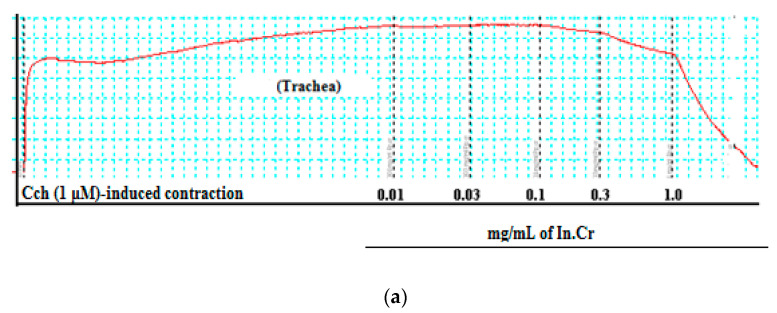

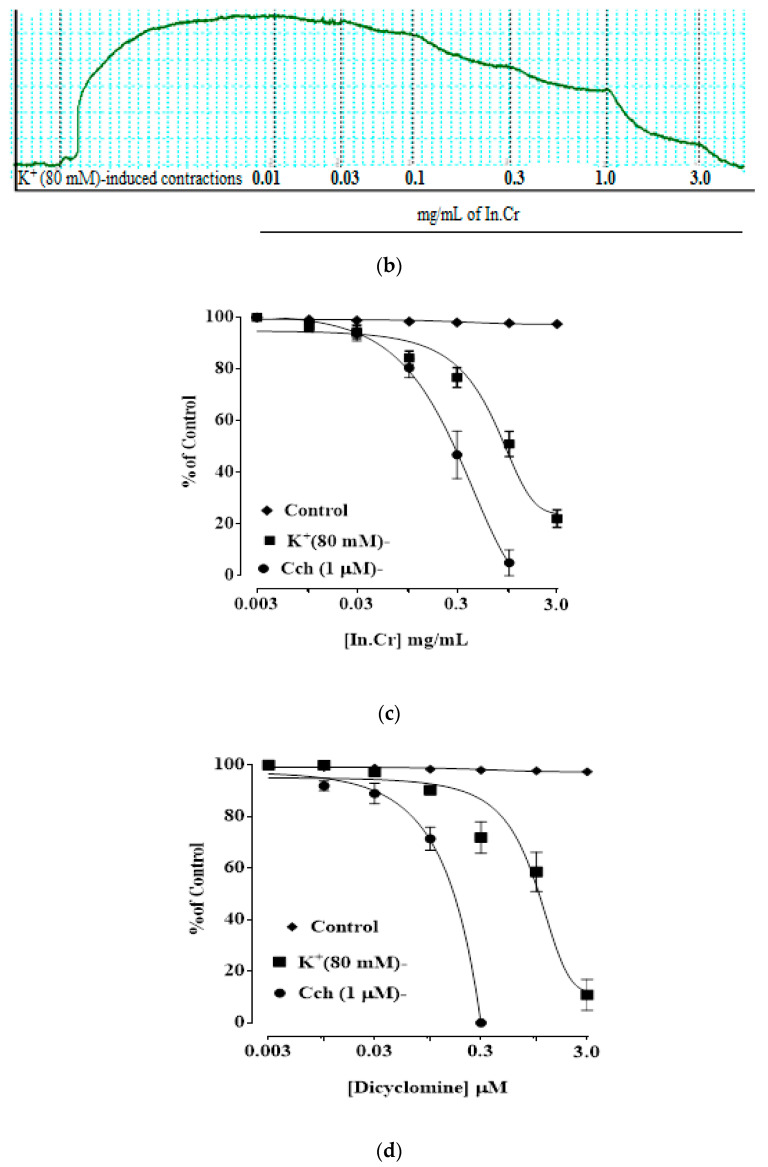
Dose dependent reduced effect (**a**–**c**) of *In.Cr* and (**d**) dicyclomine on Cch-induced slimming down on rabbit’s tracheal preparations. (*n* = 5).

**Figure 3:**
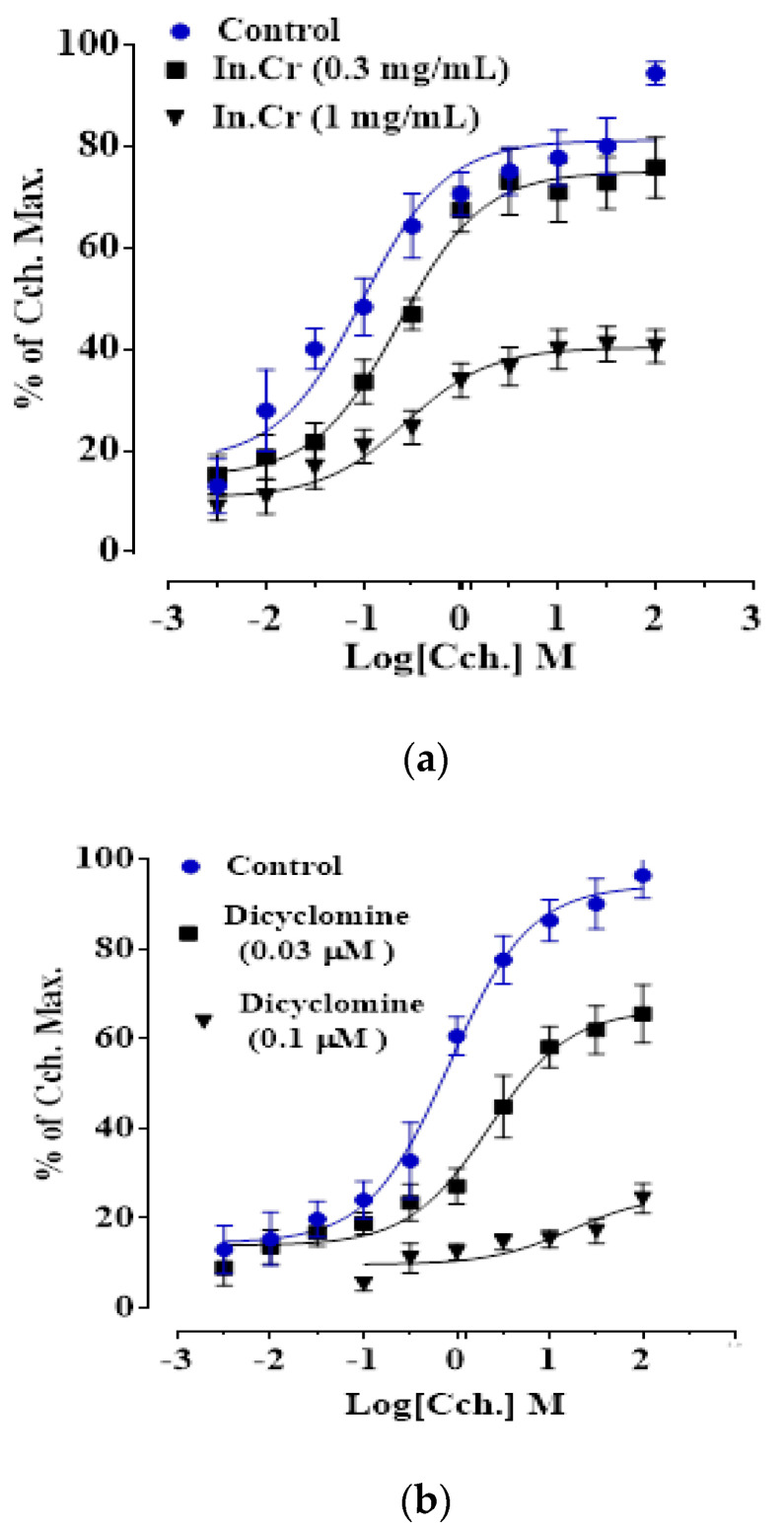
Dose-response arches of carbachol in the nonexistence and existence of the increasing extent of: (**a**) *In.Cr* and (**b**) dicyclomine. (*n* = 5).

In addition, 0.01–3.0 mg/mL of *In.Cr* expressed a concentration dependent declined effect on Cch (1 μM)- and K^+^(80 mM)-prompted contractions in an isolated rabbit’s tracheal preparation with EC_50_ values that indicated being more potent against Cch (Figure 2a–c). Likewise, dicyclomine instigated the reduction of Cch and K^+^-induced contractions through an EC_50_ value of 0.55 mg/mL and 1.07 mg/mL, respectively (Figure 2d). An application of 0.3 mg/mL produced a parallel shift to the right of the Cch-response curves with a non-inhibitory contractile response. Nonetheless, a nonparallel transference existed by reduction of the influence at an amount of 1.0 mg/mL, as dicyclomine (Figure 3).

### 3.8. In-Vivo OVA-Sensitized Asthmatic Activity

#### 3.8.1. Effects of *In*.*Cr* on OVA-Sensitized Infiltration of Inflammatory Cells

**Figure 4:**
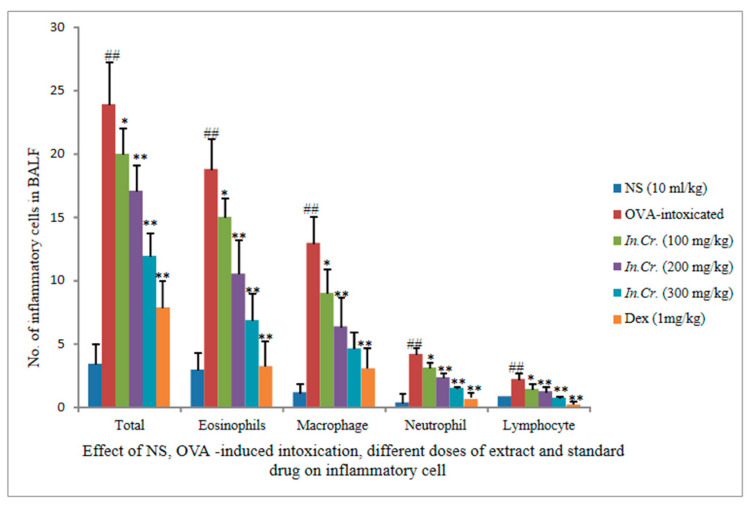
Orally *In.Cr* diminished the levels of inflammatory cells in BALF compared to OVA-challenged mice. Ovalbumin (OVA)-sensitized mice exhibited increases in Eosinophils, Macrophages, Neutrophils and Lymphocytes in contrast with the controls. However, the *In.Cr* treated mice revealed marked reductions in number of cells as compared to the OVA-challenged mice. Control group treated with normal saline (0.9%) only; OVA, OVA-challenged mice; Dex, Dexamethasone (1 mg/kg) plus OVA-challenged mice; *Ipomea nil* (100 mg/kg, 200 mg/kg, 300 mg/kg) plus OVA-sensitized mice ^#^^#^ *p* < 0.05 vs. control mice; ^#^^#^ *p* < 0.05 and ** *p* < 0.05 are more significant while * *p* < 0.01 is significant (*n* = 8).

There is a markable reduction considered to be significant in inflammatory cells count seen at 100 mg/kg. The other two doses (200 mg/kg, 300 mg/kg) of *In.Cr* indicated a more significant decline in inflammatory cells i.e., eosinophil, lymphocytes, neutrophils and macrophages as compared to an OVA-intoxicated, more significant rise in cells, mainly eosinophil, which ratifies infiltration of cells (Figure 4).

#### 3.8.2. IL-4 and IL-13

**Figure 5:**
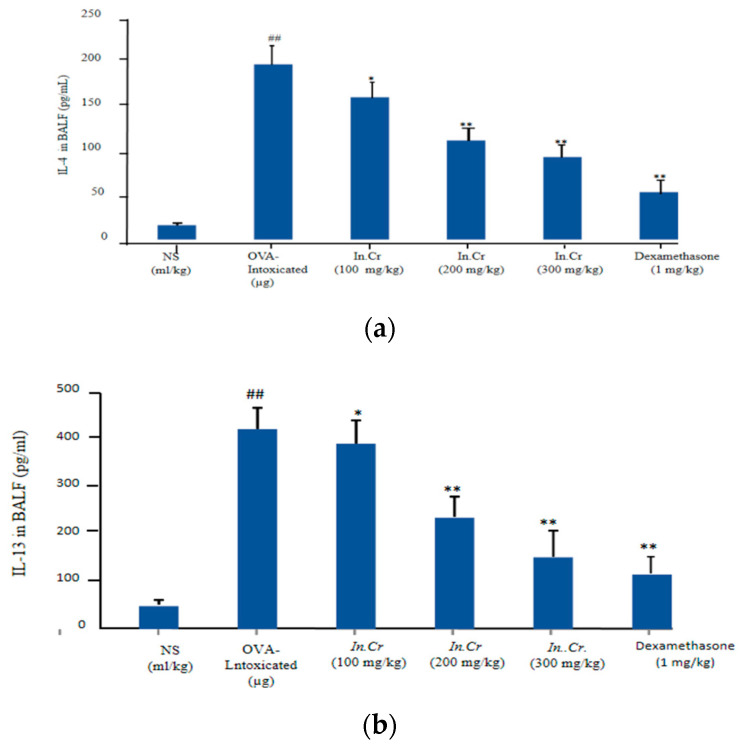
(**a**) and (**b**). *In.Cr* significantly decreased the levels of Interleukins (IL-4 and IL-13) in the BALF compared to OVA-treated mice. The ovalbumin (OVA)-treated mice unveiled rises in cytokines i.e., IL-4 as well as IL-13 compared to the controls. ^#^^#^ *p* < 0.05 and ** *p* < 0.05 are more significant while * *p* < 0.01 is significant (*n* = 8).

*In.Cr* (100 mg/kg, 200 mg/kg and 300 mg/kg) displayed a significant fall in interleukins levels compared to the OVA-intoxicated group (significant surge in number of cells; particularly, IL-4 was established (Figure 5a,b).

#### 3.8.3. Interferons (INF-γ)

**Figure 6:**
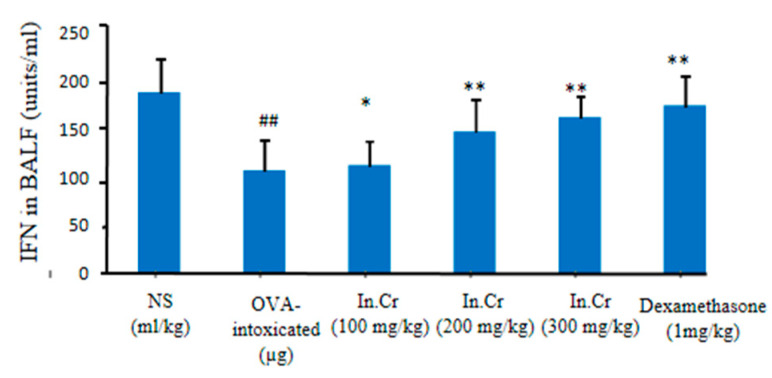
*In.Cr* more significantly boosted the levels of Interferons at 300 mg/kg by oral gavaging. The other doses of *In.Cr* also showed very effective results as expressed in the figure. The ovalbumin (OVA)-challenged group presented significant shrinkage in the levels of Interferon. ^#^^#^ *p* < 0.05 and ** *p* < 0.05 are more significant while * *p* < 0.01 is significant (*n* = 8).

Significant escalation was observed in the interferon (IFN-γ) levels at 100 mg/kg, 200 mg/kg and 300 mg/kg of *In.Cr* compared to the model (significant diminution in Interferons) (Figure 6).

#### 3.8.4. IgE

**Figure 7:**
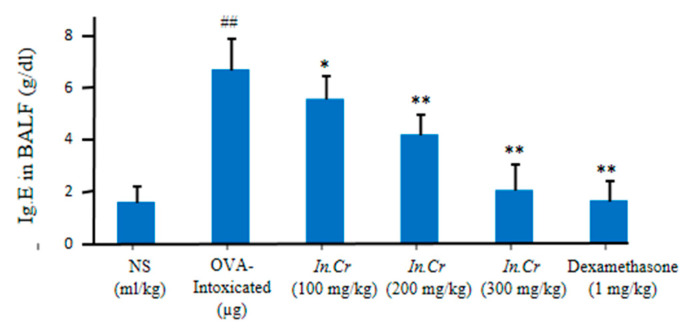
Per oral administration of *In.Cr* significantly diminished (*p* < 0.05) the ranks of IgE antibody at 100 mg/kg. *In.Cr* at higher doses more significantly ameliorated IgE, even though the ovalbumin (OVA)-tested group had significant escalation in the points of antibodies. ^##^ *p* < 0.05 and ** *p* < 0.05 are more significant while * *p* < 0.01 is significant (*n* = 8).

Furthermore 100 mg/kg, 200 mg/kg and 300 mg/kg of *In.Cr* indicated significant fall in the levels of immunoglobulin IgE antibody than OVA-intoxicated group, in which a significant increase in the number of IgE antibodies was observed, as described in Figure 7.

#### 3.8.5. Inflammation Scores

**Figure 8:**
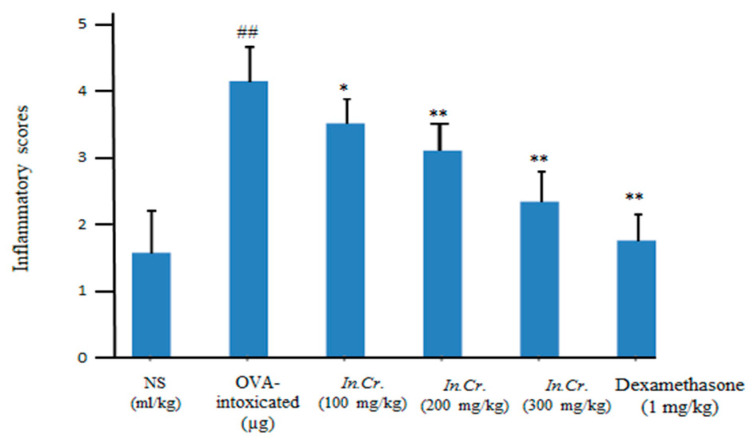
Significant reduction (*p* < 0.05) of *In.Cr* for inflammatory marks at 100 mg/kg, instead (*p* < 0.01) at 200 mg/kg in addition to 300 mg/kg. The model group displayed significant escalation in inflammation. ^#^^#^ *p* < 0.05 and ** *p* < 0.05 are significant while * *p* < 0.01 is more significant (*n* = 8).

Dose dependent significant amendment in the inflammation of OVA-sensitized allergic asthma was detected with oral gavage of *In.Cr* at 100 mg/kg, 200 mg/kg as well as 300 mg/kg compared to the intoxicated group while standard as well as control groups also displayed a decrease in inflammation scoring values (Figure 8). The analyzing parameters were 0 = normal,1 = mild, 2 = very mild, 3 = severe, and 4 = very severe.

## 4. Discussion

Medicinal plants are being widely consumed globally as a supplier of significant remedial agent for the principal health benefits [34]. *Ipomoea nil* has a folkloric temperament to generate beneficial effects in the management of numerous complaints concerning the gastrointestinal as well as the respiratory system. It also possesses the anti-bacterial, enzyme inhibition, antiemetic, hepatoprotective and antipyretic activity [13,14,16,35]. On the basis of previous concepts as well as the usage of this plant, this work commenced for the logical substantiation for these folkloric statements through an assessment of the probable mechanism(s) of action.

Preliminary phyto-investigation revealed that the *In.Cr* contains alkaloids, tannins, anthraquinons, coumarins, saponins and flavonoids as previously reported by [15], playsan anti-inflammatory as well as an antioxidant role [27,36]. Formation of free radicals (“reactive oxygen species”) via oxidative stress is involved in various un-fine conditions like ALI, asthma [37], respiratory cancer, neurodegenerative disorder, immune defects, heart diseases as well as various other ailments. The antioxidant assay directly links to the scavenging of free radicals. To check out the antioxidants’ status, TPC and TFC were executed that ranges from 96.2 ± 3.09 to 115.5 ± 1.02 mg GAE/g ofdry weight of the sample and 43.01 ± 2.12 to 50.44 ± 1.02 mg QE/g of the dry weight of the sample, respectively, which expressed the presence of polyphenols and flavonoids, given in Table 2. Table 1 expresses 86.28 ± 0.25% inhibition of free radicals’ capacity of *In.Cr* with IC50 (50% of maximum inhibitory concentration) value 17.22 ± 0.56 vs. quercetin (standard) with % inhibition value of 90.25 ± 0.99 with IC_50_ 17.47 ± 0.15 via DPPH method. The DPPH scavenging influence of *In.Cr* might be due to an elevated concentration of tannins as Refs. [38,39] reported that tannins and polyphenolics exhibit more DPPH scavenger action toward radicals. Phenolic as well as flavonoid compounds undertake as reducing agents, free radical hunters, and quenchers of single molecules of oxygen creation [40]. Furthermore, phenolic acids and flavonoids components play chief roles in the control of acute and chronic diseases including respiratory infirmities like asthma, emphysema and ARD [41,42].

HPLCis a multipurpose technique that is widely used to isolate, characterize and quantify subordinate metabolites in plant extracts [43], primarily flavonoids, phenol compounds, steroids and alkaloids [44,45]. HPLC analysis of *In.Cr* expressed the peaks and quantification of antioxidants: polyphenolics and flavonoids; gallic acid, ferulic acid, syringic acid, benzoic acid, *p* and m-coumaric acid, vanillic acid as well as chlorogenic acid and kaempferol, which inhibit enzymes, inflammation [46] and oxidative stresses on the cellular level and scavenge the cells from free radicals by retarding oxidative products’ formation [47]. The quantities along with peaks of antioxidants are expressed in chromatograms in Figure 1a,b. Kaempferol, a plant flavonoid, and its glycosylated derivative, kaempferol-3-O-rhamnoside (K-3-rh), have the potential for anti-inflammatory, antioxidant, and anti-asthmatic effects in an asthma model mouse by diminishing inflammatory cells, IL-4 and IL-13, TNF-α as well as IgE immunoglobulin [32]. Polyphenols have great potential to reduce severe asthma via ameliorating inflammation and oxidation [48,49,50,51,52,53,54].

Calcium channel blockers (CCCs) are effective for asthma [30] having bronchodilatory action. To assess the Ca^2+^ channel blockade, *In.Cr* was tested on the contractions prompted by K^+^(80 mM), and rabbit tracheal preparations started to be unperturbed by *In.Cr.* It is known that K^+^(80 mM) is familiarized to induce contractions of smooth muscle by activation of voltage-dependent L-type Ca^2+^ channels (VDLCs); as a result, generation of extracellular Ca^2+^ invasion [55] and a constituent instigating inhibition of high K^+^-made contraction are measured as Ca^2+^ influx blockers [56];thus, causing a decline in cytosolic calcium originates a decrease in calcium binding to calmodulin. Thecomplex of calcium calmodulin (CCC) ought to trigger a myosin kinase sequence using the secondary phosphorylation of light fetters. Hence, breakage in the cascade results via inhibition of calcium. The hypothesis was certain as *In.Cr* shifted to the right of the Ca^2+^ response curve analogous to that of verapamil [57]. The *In.Cr* caused relaxation of Cch-induced contractions at a lower quantity but K^+^-induced contractions at a greater concentration, proposing the concomitance of anticholinergic plus Ca^2+^ blocking properties, as of dicyclomine [58]. *In.Cr* generated curves to the right and Refs. [30,59] describe how CCBs may illuminate the worth of the plant in different respiratory complications i.e., asthma. A number of scientists have proposed that augmented Ca^2+^ signaling is involved on the basis of obstructive airway diseases, accompanied with airway hyper reactivity [60].

A few studies have also presented that smooth muscle of airways from asthma and COPD sufferers respond with increased maximal force to contractile stimulation invitro [61,62], while hyper responsive animals showed elevated Ca^2+^ responses against less reactive rats [63,64]. As a result, intrinsic irregularities interrelated to cholinergic hyperactivity may exist in asthmatics and COPD patients. There is an increase in eosinophilic airways among inflammatory cells in mild-severe asthma [65,66]. We observed escalation infiltration of inflammatory cells i.e., eosinophils, neutrophils, macrophages as well as lymphocytes in BALF of the OVA-intoxicated group but significant (*p*-value less than 0.05) fall infiltration of cells in airways in orally *In.Cr* treated animals, in a dose dependent way (100–300 mg/kg). From a pathological point of view, inflammatory score was also lower in *In.Cr* treated mice than in the model group, which shows severe asthmatic response (Figure 8).

(IFN-γ) is a creation of T_H1_ cells that exerts inhibitory effects on T_H2_ cell differentiation. In the pathogenesis of allergic asthma, the involvement of these cytokines is vitally considered. In this context, IFN-γ production in asthma has been observed to be decreased in *In.Cr* treated mice compared to the OVA-intoxicated group, and this reduces their capacity to inhibit *IgE* synthesis as well as allergic inflammation [67,68]. The obtained facts further strengthen the notion that the pathogenesis of the lesions of asthma, and especially of AHR, involves a co-operative interaction between Th2 and Th1 cytokines [69]. In this case, *In.Cr* clearly improved IFN-γ (Figure 7) compared to the results of the intoxicated group, which can be more helpful to treat asthma or other respiratory problems. *IgE* acts as a central player in asthmatic events in patients, targets two main receptors (i) FcεRI (ii) CD23 and results in severity [10] of acute allergic conditions, especially of airways. Orally, 100 mg/kg of *In.Cr* significantly (*p* < 0.05) lessened immunoglobulin *IgE* antibody, while 200 plus 300 mg/kg expressed more significant (*p* < 0.01) attenuation (Figure 8). The ovalbumin (OVA)-treated animals showed a significant increase in immunoglobulin (*IgE*) antibody.

Interleukins are responsible for several biological matters. The signaling occurs via the IL-4Rα receptor and has consequences for the stimulation of STAT6 and Insulin Receptor Substrate fragments. IL-4Rα signaling subsidizes towards cell-mediated along with humeral parts of allergic inflammation, discharge IL-4 plus IL-13 in the airline, intensifying tissue reactions and performing a crucial part in production i.e., inflammation of eosinophilic, airway hyper-responsiveness, too much mucus production, as well as epithelial fibrosis [70,71].The anti-inflammatory actions of flavonoids involve the reduction in synthesis and slow down the activities of some pro-inflammatory mediators such as cytokines, eicosanoids, adhesion molecules as well as C-reactive protein [72]. Cysteinylleukotrienes, i.e., C4, D4 and E4, were initially considered as powerful vasoconstrictors (asthmatic). At present, it is also recognized that allergic rhinitis, through escalation of tenderness, capillary permeability and mucus exudation, facilitated by binding of the cysteinylleukotrienes to the cysLT receptor; thus, antagonism of this receptor is essential to manage allergic rhinitis as well as asthmatic conditions [73,74,75]. *In.Cr* significantly improved interleukins-4 and 13 at all the given doses compared to in OVA-prompted asthmatic mice, expressing a significant increase in interleukins (Figure 5a,b).

Moreover, *In.Cr* demonstrated being a very good cholinesterase inhibitor (acetyl- and butyryl-) (Table 3) and plays a substantial role in modifying the acetylcholine level at synaptic cleft. Muscarinic enzyme inhibition may reduce inflammation as well as sepsis by generating an anti-inflammatory pathway [76,77]. Meanwhile, inhibitors of cholinesterase play a significant role in managing Alzheimer’s disease and myasthenia gravis [58,78,79]. Hereafter, an antioxidant and anti-inflammatory summary of *In.Cr* will possibly have a dynamic impact on the modification of pathology of declared respiratory diseases.

## 5. Conclusions

It can be inferred from the outcomes of research that the bronchodilator and anti-asthmatic assays may be endorsed due to the obstruction of Ca^2+^ channels and/ muscarinic enzymes, deteriorating ROS-related processes as well as the existence of antioxidants i.e., polyphenols and flavonoids, which may be responsible for systematic mechanistic circumstances to corroborate ethnopharmacological privileges for *Ipomoea nil* in managing respiratory problems i.e., COPD and asthma.

## Figures and Tables

**Table 1:** IC_50_ data.

Sample	%Inhibition	IC_50_ (mol/L)
*In.Cr*	86.28 ± 0.25	17.22 ± 0.56
Quercetin	90.25 ± 0.99	17.46 ± 0.15

**Table 2:** Total phenolic and flavonoid contents of *In.Cr*.

S. No.	*In.Cr*	TPC (mg GAE/g of *In.Cr*)	TFC (mg QE/g of *In.Cr*)
1.	1 mg/mL	96.2 ± 3.09	43.01 ± 2.12
2.	1 mg/mL	107.8 ± 1.29	46.02 ± 1.10
3.	1 mg/mL	115.5 ± 1.02	50.44 ± 1.06

Each value describes the mean of three replications ± standard deviation (Mean ± SEM).

**Table 3:** Inhibitory profile of *In.Cr* oncholinesterases’ enzyme actions.

Enzymes	Test Substances	%Inhibition	IC_50_ (mol/L)
Acetylcholinesterase	*In.Cr*	64.67 ± 0.46	0.60 ± 0.67
	Eserine	90.37 ± 1.07	0.06 ± 0.002
Butyrylcholinesterase	*In.Cr*	70.65 ± 0.50	1.5 ± 0.04
	Eserine	80.85 ± 1.10	0.30 ± 0.003

Each value describes the mean of three replications ± standard deviation (Mean ± SEM).

## Data Availability

Not applicable.
